# p40 as a Basal Cell Marker in the Diagnosis of Prostate Glandular Proliferations: A Comparative Immunohistochemical Study with 34betaE12

**DOI:** 10.1155/2015/897927

**Published:** 2015-03-08

**Authors:** Hermann Brustmann

**Affiliations:** Department of Pathology, Landesklinikum Baden-Moedling, Wimmergasse 19, 2500 Baden, Austria

## Abstract

Immunohistochemistry is important for the accurate diagnosis of basal cells in atypical glandular proliferations of the prostate. p40, an isoform of p63, may be an adjunct to a marker panel in this setting. Biopsies of 68 patients were analyzed by immunohistochemistry using antibodies to 34betaE12 and p40. Basal cell staining was classified as negative, partial (<60%), or diffuse (≥60%); irregular staining was defined as discordant staining patterns. In acinar proliferations (*N* = 41), partial staining for both markers was seen in 42%, and diffuse staining in 46% of reactive cases. An irregular reactivity was noted in one case only (2%). Finally, these lesions were signed out as benign. Acinar proliferations negative for both markers and limited amount of glands (≤4) were termed atypical small acinar proliferations (ASAP). Out of six PIN lesions two cases showed partial, three cases showed diffuse reactivity for both markers, and one case was stained irregular. All cases diagnosed as prostate carcinomas (*N* = 20) had no evidence of basal cell staining for neither of the markers. p40 expression is closely correlated to 34betaE12 with respect to demonstration of basal cells of prostate glands and may provide further information on the dignity of glandular proliferations of the prostate.

## 1. Introduction

Immunohistochemistry is an important tool in the differential diagnosis of prostate cancer. In particular, this is true in many instances of prostate needle biopsies presenting with limited amounts of atypical glandular proliferations. Small atypical foci may be challenging for the diagnosing pathologist by raising a suspicion for malignancy [1]. The identification of basal cells is considered helpful in excluding a diagnosis of prostate adenocarcinoma [2]. There is a small number of immunohistochemical markers that have been shown valuable in the demonstration of basal cells in prostate glandular tissues. The antikeratin antibody 34betaE12 (also known as keratin 903) is well recognized in this setting [2, 3]. Another standard marker of basal cells of the prostate gland is p63 [4].

p63 is normally expressed in the basal cell layer of stratified epithelia like squamous or urothelial tissues as well as in basal cells of prostatic epithelia, myoepithelial cells of breast and salivary glands, trophoblasts, and thymic epithelial cells [5]. It consists of several isoforms. They fall into two major groups: TAp63 and ΔNp63. The latter was noted as the predominant p63 transcript in squamous lung cancers and carcinomas of other sites. The antibody designated as p40 recognizes exclusively ΔNp63 and not TAp63 [5]. In the prostate gland recent work has shown that p40 stains prostatic basal cells as reliable as p63 in most cases. Aberrant staining of tumor cells was seen more rarely with p40 than with p63 [4]. p63 immunostaining has been compared with 34betaE12 previously [6]. These authors concluded that for diagnosing prostate carcinoma in needle biopsies p63 is as specific and sensitive as 34betaE12 and therefore can be used as a complementary basal cell-specific stain in difficult cases. Others noted that a basal cell cocktail consisting of 34betaE12 and p63 improves the detection of prostate basal cells [7, 8].

Since p40 is just the ΔNp63 isoform of p63, it seems justified evaluating its value as a marker on its own different diagnostic settings. This study compared the performance of a p40 versus a 34betaE12 antibody in a series of prostate needle biopsies to test whether p40 is another diagnostically valuable basal cell marker in prostate glands to differentiate atypical glandular proliferations from prostate cancers and to determine potential limitations of this staining protocol.

## 2. Material and Methods

All cases of prostate specimens diagnosed at our institution between October 2012 and December 2013 were retrieved from the department's files. Among a total of 338 patients 62 cases with needle biopsies and 6 cases with TURP (transurethral resection of the prostate) investigated by 34betaE12 and p40 immunohistochemistry at the time of histopathologic work-up were identified and retrospectively analysed. Patients' age ranged from 43 to 82 years, with a median of 69 years. All original hematoxylin-and-eosin (H&E) and immunohistochemically stained sections as well as the clinical histories were reviewed. The specimens were fixed in formalin and embedded in paraffin. Formalin fixation did not exceed 24 h. The study was approved by the local ethical committee (# GS4-EK-4/270/2014).

Step sections of the same paraffin-embedded tissues as those used for the H&E -stained sections were used for immunohistochemistry, applying the same staining protocol for all cases. They were cut at 3 *μ*m. Subsequently, the sections were deparaffinized in xylene and rehydrated via graded ethanol. A standard immunohistochemical technique was performed using a Ventana BenchMark XT immunostainer with a ready-to-use monoclonal mouse anti-human 34betaE12 antibody (code IR051, DakoCytomation, Denmark, incubation time 32 minutes), and a prediluted ready-to-use rabbit polyclonal antibody to p40 (catalog no. API 3030 AA, Biocare Medical, Concord, CA, incubation time 40 minutes). Heat epitope retrieval as provided by the immunostainer was done in a TRIS based buffer supplied by the manufacturer (CCl cell conditioning solution, Ventana Medical Systems, Illkirch, Cedex, France) at pH8 for 30 minutes for all antibodies used. The enzymatic reactivity was visualized by 3-3 diaminobenzidine (DAB). Cases of a normal prostatic tissue with periglandular basal cells retrieved from a prostatectomy specimen and a normal epidermis served as external positive control, indicative for strong staining intensity. For negative controls serial sections of the same specimens were used, omitting the primary antibody from the staining protocol and substituting it by commercially available mouse nonimmune IgG serum. The immunohistochemical slides were evaluated and interpreted without knowledge of the clinical data in a blinded manner.

Immunoreactivity was scored by screening the slides at low power for any staining of basal cells; higher magnification (×100) was used to determine staining pattern which was evaluated semiquantitatively as follows: no staining (0%), partial staining (<60%), diffuse staining (≥60%), and compared casewise. This method of quantification is similar to protocols applied for 34betaE12 and p63 previously [4, 8].

The statistical tests were calculated with GraphPad Prism statistical analysis software (GraphPad Software, version 6.0, San Diego, CA), applying chi-square test or Fisher's exact test for different reactivity patterns and groups of patients, and column statistics for patients' ages (median, standard deviation (SD), minimum, maximum, and range). A *P* value of <0.05 was considered significant.

## 3. Results

At the time of diagnosis 34betaE12 and p40 immunostaining was employed to study glandular proliferations of the prostatic tissue present in small amounts and felt to be “atypical.” In particular this was true for small acinar glands raising some suspicion for acinar carcinoma. The differential diagnosis for these cases encompassed lobular and partial atrophy, postatrophic hyperplasia, adenosis, normal structures like verumontanum glands, and inflammation associated changes. In some lesions suspicious for high grade prostatic intraepithelial neoplasia (PIN) staining was done to differentiate them from cribriform adenocarcinoma. This setting occurred in PINs with somewhat irregular outlines raising the question of cribriform carcinoma or to determine the Gleason grade in acinar carcinomas associated with such glands.

Staining for 34betaE12 was cytoplasmic, p40 staining was nuclear. A fine granular cytoplasmic p40 and 34betaE12 immunoprecipitate was noted in some cases of glandular epithelial cells or stromal cells and considered nonspecific, in occasional association with mild crush artifacts. Benign prostate glands showed strong and diffuse basal cell staining with both markers. Three main categories of prostate tissues were distinguished. Group 1 consisted of acinar proliferations (*N* = 42), group 2 of prostatic intraepithelial neoplasia (high grade, *N* = 6), and group 3 of adenocarcinomas (*N* = 20). The latter displayed Gleason grade 3+3 (*N* = 11), 3+4 (*N* = 4), 4+3 (*N* = 2), and 4+4 (*N* = 3). One case of group one had to be excluded from this study since there was not sufficient glandular tissue left in deeper step sections performed for immunohistochemistry.

**Figure 1:**
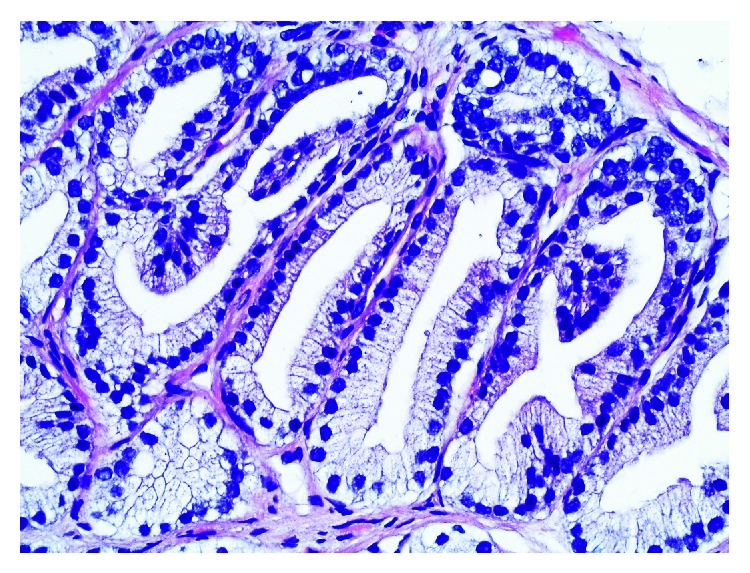
A case showing tightly clustered glands, with few intervening stomata. This architecture raised some suspicion, and the cytology advocates for a benign lesion. After basal cell staining a hyperplastic nodule was diagnosed (H&E, ×400).

**Figure 2 fig2:**
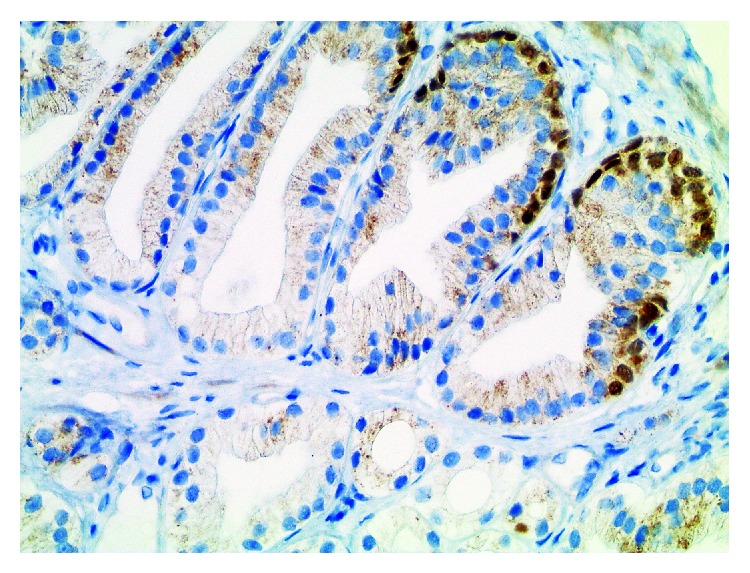
Same case as shown in Figure 1. There is evidence of basal cells on p40 immunohistochemistry in a partial pattern. Immunoreactivity is nuclear (×400).

**Figure 3:**
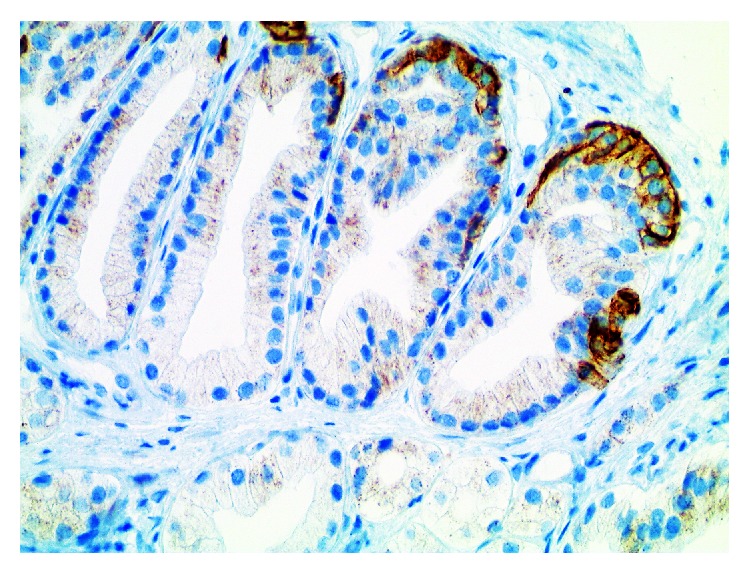
Same case as shown in Figure 1. There is evidence of basal cells on 34betaE12 immunohistochemistry in a partial pattern. Immunoreactivity is cytoplasmic (×400).

**Figure 4:**
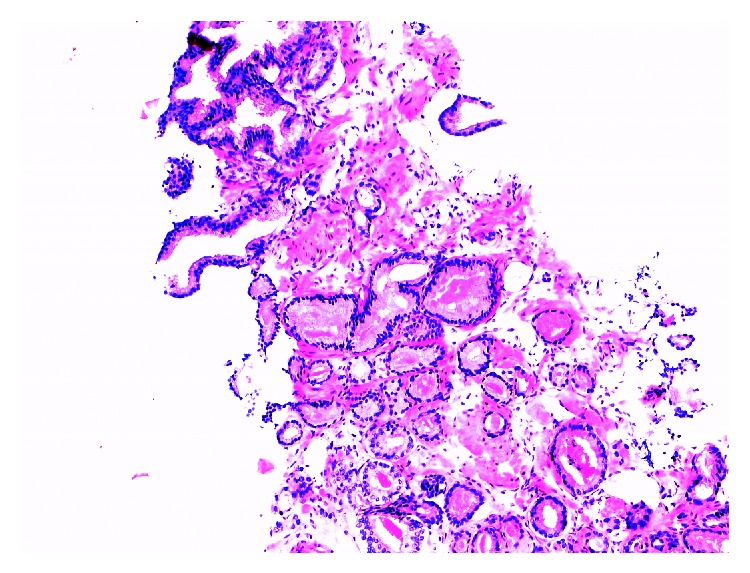
Well-differentiated acinar glands in a haphazard pattern are considered suspicious for carcinoma. The cytoplasm is pale and abundant; there is some nuclear atypia, and intraluminal eosinophilic bodies are occasionally seen. After immunohistochemistry for basal cells this focus was signed out as acinar adenocarcinoma: Gleason 3+3 (H&E, ×100).

**Figure 5 fig5:**
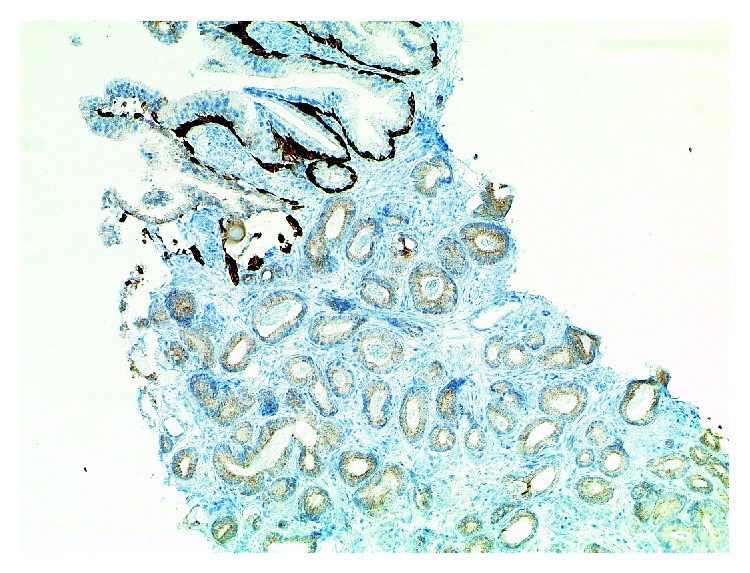
Same case as shown in Figure 4. Complete lack of basal cells in the atypical glandular proliferation is noted on 34betaE12 immunohistochemistry. Basal cells are evident in adjacent noninfiltrative glands displaying features of intraepithelial neoplasia/PIN (×100).

**Figure 6:**
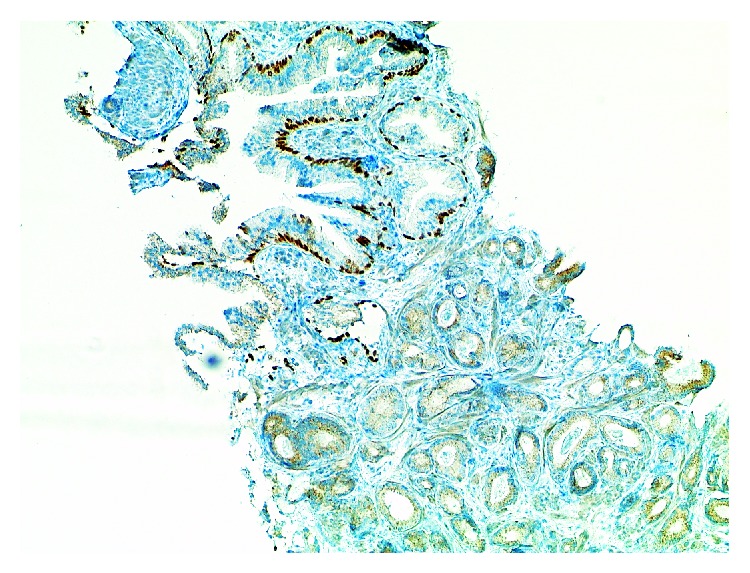
Same case as shown in Figure 4. Complete lack of basal cells on p40 immunohistochemistry. There is some weak nonspecific cytoplasmic staining of tumor cells. Basal cells are evident in adjacent noninfiltrative glands displaying features of intraepithelial neoplasia/PIN (×100).

In group 1, four cases (10%) of atypical glandular proliferations were completely negative for basal cell staining with both markers; due to the limited amount of glands (≤4) these cases were termed atypical small acinar proliferations (ASAP). 17 cases (42%) showed partial and 19 cases (46%) diffuse staining patterns. An irregular pattern was recorded in one case (2%) with a diffuse staining for 34betaE12 and partial staining for p40. Casewise comparison in partially and diffusely stained cases revealed identical quantities of immunoreactive basal cells for both markers (Figures 1 [Fig fig2]–3). In PINs there were no cases negative for basal cells (Figures 4 [Fig fig5]–6). Partial staining was seen in two cases and diffuse staining in three cases. One case belonged to the category of irregular staining, with diffuse 34betaE12 reactivity. There was no statistical difference between staining patterns for 34betaE12 and p40 (*P* = 1.0000, chi-square test). All cases with evidence of basal cells were signed out as benign.

All cases signed out as carcinomas were completely negative for both markers (Figures 4–6). The biopsy diagnoses were confirmed as carcinomas in subsequent prostatectomy specimens. Staining between benign glandular proliferations and prostate cancers differed significantly (*P* < 0.0001, Fisher's exact test).

## 4. Discussion

In this study a close correlation of prostate gland basal cell staining for antibodies to 34betaE12 and p40 was noted in the vast majority of cases, with concordant staining patterns. Irregular staining was observed in one case only. The findings presented herein thus indicate that p40 may be used to label basal cells of prostate glands and may be included in a marker panel to demonstrate the latter in diagnostic difficult settings. This situation emanates primarily from needle biopsies with a limited amount of suspicious glands. Looking at such glands on H&E stained sections we use an approach of “favoring benign” or “favoring malignant.” This first evaluation is based on previously defined criteria for minimal carcinoma in prostate biopsy specimens [9]. The most important features are nucleomegaly, infiltrative growth pattern, intraluminal secretions, prominent nucleoli, association with high grade PIN, amphophilic cytoplasm, nuclear hyperchromasia, absence of basal cells, and intraluminal crystalloids [10]. An infiltrative growth pattern is an important feature in Gleason 3 acinar minimal carcinomas with a dimension of <1 mm recognized in needle biopsies, displaying a few malignant glands between benign glands. In contrast to PIN lesions, high grade carcinomas of Gleason scores 4 and/or 5 show a ragged pattern of invasion; however, it is challenging to differentiate atypical high grade PINs and carcinomas in some instances on needle biopsies [11]. Evaluation of nuclear atypia may be difficult in a setting of rather well-differentiated Gleason 3 acinar glands [9, 10].

The appreciation of basal cells in prostatic glands may be problematic in some instances on H&E staining; they may seem lacking, or fibroblasts from the periglandular stroma closely adjacent to the epithelial cells may mimic basal cells. Immunostains for basal cell markers like 34betaE12, p63, or p40, respectively, may assist in the diagnosis in such instances. However, such negative stains are not necessarily indicative for malignancy. In an early study, Hedrick and Epstein [2] noted a complete lack of basal cells by keratin 903 (34betaE12) in 8% of atrophic glands, 12% of glands in basal cell hyperplasia, and 39% of glands in atypical adenosis; however, all grades of adenocarcinoma lacked any immunoreactivity. Others found that a fragmented basal cell layer is characteristic of atypical adenomatous hyperplasia (adenosis), with a lack of a basal cell layer in approximately 50% of glands [3]. Thus, evaluating immunostains for the abovementioned basal cell markers should be made with caution, and neglecting basic morphologic features of the glands under suspicion is to be avoided.

The definition of the minimal number of glands to diagnose prostatic adenocarcinoma on needle biopsy is a crucial matter. Diagnostic criteria of malignancy as described above have to be met in these glands. Additionally, these few glands must not show any evidence of basal cells by immunohistochemistry. The cut-off value in such instances considered diagnostic of carcinoma at our institute is five glands. This is in agreement with van der Kwast et al., who felt that foci comprising < 6 glands are equivocal [1]. Others concluded that three glands constituted the lowest numerical cut-off [12].

In agreement with recent literature it is evident that immunohistochemistry is frequently used in prostate biopsy practice to establish or confirm a limited cancer diagnosis [13]. Besides the use of a racemase (AMACR), 34betaE12 and p63 are well-established markers in this setting [2–4, 13]. Since there are many entities entering the differential diagnosis of prostatic adenocarcinoma, sensitivity and specificity of such markers need to be established and, on the other hand, the immunoreactivity patterns of potential mimickers are to be characterized. Normal structures like crowded glands as well as Cowper's glands or seminal vesicles, verumontanum glands, atrophy in its variants, and postatrophic hyperplasia as well as adenosis (atypical adenomatous hyperplasia) are the probably most common conditions to be misinterpreted for adenocarcinoma, as are rarer entities like nephrogenic adenomas or mesonephric hyperplasia [14, 15]. Previously, Kahane et al. have described that the use of high molecular weight cytokeratin decreased the rate of an atypical prostate biopsy from 8.3% to 0.4% [16]. Shah et al. noted the usefulness of a basal cell cocktail consisting of 34betaE12 and p63 in the diagnosis of atypical glandular proliferations of the prostate [8]. These studies also emphasized the cost-effective improvement in analyzing difficult prostatic lesions by these markers. Single cribriform glands may pose a differential diagnosis between cribriform carcinoma and high-grade PIN. Amin et al. analyzed cribriform morphology in prostatic neoplasia using HMWKs [17]. They concluded that HMWK staining to detect basal cells in isolated cribriform glands encountered in biopsy specimens may be a useful diagnostic discriminant between high-grade PIN and prostatic carcinoma. However, they observed focal and isolated HMWK positivity amid prostatic carcinoma and, thus, emphasized that the overall pattern of staining together with morphological features is critical to exclude carcinomas [17]. The study presented herein provides evidence that p40 is a sensitive and specific nuclear marker in the basal cell detection of such atypical glands in prostate biopsies, with a high concordance with the HMWK 34betaE12, therefore offering another complementary basal cell stain.

Atypical small acinar proliferations (ASAP) are represented by a small focus of atypical glands suspicious for carcinoma that cannot be safely diagnosed as benign or malignant on H&E as well as immunostains. Features of ASAP are summarized by Schlesinger et al. [18]. In the study at hand four cases displaying acinar morphology of ≤4 glands accompanied by low grade cytological atypia and lack of evidence of any basal cells were placed into this category. There is no available recent follow-up of these cases. However, in such small lesions the lack of basal cells should not be overinterpreted, since staining may only be evident in partial patterns in many benign instances as discussed above and, thus, may be missed in single glands found on biopsy.

Recently, p40 immunostaining has gained a wide recognition in tumor diagnosis, with special regard to squamous cell and urothelial carcinomas [19, 20]. In the prostate, p40 has been shown to stain basal cells as reliable as p63, with only minor differences [4]. As also noted in the study at hand, some weak granular cytoplasmic reactivity of p40 narrows its eligibility for antibody cocktails containing AMACR [4]. Tacha et al. included three cases of prostatic tissues in their study on p40 in a large number of carcinomas of different origins and observed basal cell staining in benign prostate as well as PIN glands [19]. In the differential diagnostic workup of atypical glandular lesions of the breast it has been suggested that p40 can also be used to label breast myoepithelial cells [21].

In conclusion, this study provides evidence for the usefulness of p40 in the diagnosis of suspicious prostate glands, and for the first time a close correlation with the well-established basal cell marker 34betaE12. Still, morphological assessment of prostate glandular tissues remains the gold standard, and any immunohistochemical ancillary testing should be considered as aid to the final diagnosis only.
